# Comprehensive Dissection of Treatment Patterns and Outcome for Patients With Metastatic Large-Cell Neuroendocrine Lung Carcinoma

**DOI:** 10.3389/fonc.2021.673901

**Published:** 2021-07-08

**Authors:** David Fisch, Farastuk Bozorgmehr, Daniel Kazdal, Jonas Kuon, Laura V. Klotz, Rajiv Shah, Florian Eichhorn, Mark Kriegsmann, Marc A. Schneider, Thomas Muley, Albrecht Stenzinger, Helge Bischoff, Petros Christopoulos

**Affiliations:** ^1^ Department of Thoracic Oncology, Thoraxklinik and National Center for Tumor diseases (NCT), Heidelberg University Hospital, Heidelberg, Germany; ^2^ Translational Lung Research Center Heidelberg, Member of the German Center for Lung Research (DZL), Heidelberg, Germany; ^3^ Institute of Pathology, Heidelberg University Hospital, Heidelberg, Germany; ^4^ Department of Thoracic Surgery, Thoraxklinik at Heidelberg University Hospital, Heidelberg, Germany; ^5^ Translational Research Unit, Thoraxklinik at Heidelberg University Hospital, Heidelberg, Germany

**Keywords:** large-cell neuroendocrine lung carcinoma, immunotherapy, platinum chemotherapy, local therapies, overall survival, *de novo* metastatic, secondary metastatic

## Abstract

**Background:**

Large-cell neuroendocrine lung carcinoma (LCNEC) is a rare pulmonary neoplasm with poor prognosis and limited therapeutic options.

**Methods:**

We retrospectively analyzed all patients with metastatic LCNEC in the records of a large German academic center since 2010.

**Results:**

191 patients were identified with a predominance of male (68%) smokers (92%) and a median age of 65 years. The single most important factor associated with outcome was the type of systemic treatment, with a median overall survival (OS) of 26.4 months in case of immune checkpoint inhibitor administration (n=13), 9.0 months for other patients receiving first-line platinum doublets (n=129), and 4.0 months with non-platinum chemotherapies (n=17, p<0.01). Other patient characteristics independently associated with longer OS were a lower baseline serum LDH (hazard ratio [HR] 0.54, p=0.008) and fewer initial metastatic sites (HR 0.52, p=0.006), while the platinum drug type (cisplatin *vs.* carboplatin) and cytotoxic partner (etoposide *vs.* paclitaxel), patients’ smoking status and baseline levels of tumor markers (NSE, CYFRA 21-1, CEA) did not matter. 12% (23/191) of patients forewent systemic treatment, mainly due to tumor-related clinical deterioration (n=13), while patient refusal of therapy (n=5) and severe concomitant illness (n=5) were less frequent. The attrition between successive treatment lines was approximately 50% and similar for platinum-based *vs.* other therapies, but higher in case of a worse initial ECOG status or higher serum LDH (p<0.05). 19% (36/191) of patients had secondary stage IV disease and showed fewer metastatic sites, better ECOG status and longer OS (median 12.6 *vs.* 8.7 months, p=0.030). Among the 111 deceased patients with palliative systemic treatment and complete follow-up, after exclusion of oligometastatic cases (n=8), administration of local therapies (n=63 or 57%) was associated with a longer OS (HR 0.58, p=0.008), but this association did not persist with multivariable testing.

**Conclusions:**

Highly active systemic therapies, especially immunotherapy and platinum doublets, are essential for improved outcome in LCNEC and influence OS stronger than clinical disease parameters, laboratory results and other patient characteristics. The attrition between chemotherapy lines is approximately 50%, similar to other NSCLC. Patients with secondary metastatic disease have a more favorable clinical phenotype and longer survival.

## Introduction

Large-cell neuroendocrine lung carcinomas (LCNEC) include approximately 3% of pulmonary malignancies and combine characteristics of the neuroendocrine and non-small-cell (NSCLC) histotypes (1). Their cells typically have a size larger than three times the diameter of resting lymphocyte, abundant cytoplasm, prominent nucleoli, and display organoid nesting, trabecular growth, rosette-like structures and/or nuclear palisading, along with positivity for at least 1 of the neuroendocrine markers synaptophysin, chromogranin A, and CD56 (2). Up to 20% of cases show a mixed histology with an additional squamous, adenocarcinoma or not-otherwise-specified NSCLC component, but this cannot be reliably assessed based on the small biopsies routinely used in the metastatic setting (3). The typically high proliferation rate (>11 mitoses/10 high-power fields or Ki67 >50%) is associated with an aggressive clinical course and poor prognosis (4–6).

Therapeutic progress is particularly difficult for LCNEC. Routinely treatable genetic alterations, such as *EGFR* mutations or *ALK* translocations, are exceedingly rare, so that tyrosine kinase inhibitors (TKI) and other targeted drugs have a very limited role (7, 8). In addition, due to the rarity of these patients, there are no clinical trials that define the optimal therapy for either localized or advanced disease (9). Treatment decisions are based on a few small retrospective series and rely on the recommendations for other NSCLC or SCLC. In case of chemotherapy doublets, the choice of the platinum partner between etoposide and NSCLC cytostatics (mainly paclitaxel, gemcitabine, and pemetrexed) has been controversial in the literature (10–12). Interestingly, more recent studies have suggested differential sensitivity according to presence of an SCLC or NSCLC-typical molecular profile (i.e. *TP53* and *RB1 vs. KRAS* and *STK11*/*KEAP1* alterations), however, efficacy remains limited with a median overall survival (OS) invariably shorter than 10-12 months (8, 13, 14). Administration of immune checkpoint inhibitors appears to prolong survival in some retrospective series (15–18), however, the overall impact in the real-world setting is challenging to assess, due to the considerable clinical heterogeneity of this rare disease. Aim of the current study was to thoroughly dissect of LCNEC management across the entire clinical spectrum of the disease.

## Materials and Methods

### Study Population and Study Endpoints

This retrospective study included all patients with metastatic LCNEC identified in the records of our institution between 2010-2020. Tumors with a small-cell component, tumors with large-cell histology lacking evidence of neuroendocrine differentiation, as well as early-stage tumors without relapse after definitive local treatment were excluded. Two main types of analyses were performed: i) patient progression-free (PFS) and overall survival (OS) according to various clinical and laboratory parameters at baseline, as well as the clinical presentation and association with PFS/OS of secondary *vs. de novo* stage IV disease were analyzed in the entire study population; ii) administration of subsequent systemic treatment and the association of palliative local therapies with PFS/OS were analyzed in the subset of deceased patients, who received palliative systemic treatment and for which complete follow-up until death was available.

### Data Collection and Statistical Analysis

Histologic diagnosis was performed on tissue specimens according to the criteria of the current WHO classification (2015) for lung cancer, and tumors were screening for actionable mutations based on indication by the treating oncologists (mainly light/never-smoking status according to the current guidelines) using combined DNA/RNA next-generation sequencing, as described previously (3, 19, 20).

All 191 patients showed positivity for at least one of the immunohistochemical neuroendocrine markers (chromogranin A, synaptophysin, CD56) with a cut-off of at least 10% staining tumor cells. The PD-L1 tumor proportion score (TPS) was determined by immunohistochemistry using the SP263 clone (Ventana/Roche, Mannheim, Germany). Clinical data were systematically collected from the patients’ records with a cut-off on December 31st 2020. Comorbidities were summarized using the Simplified Comorbidity Score (SCS), a validated predictor of mortality in NSCLC (21). The progression date under systemic treatment was verified by the investigators with review of radiologic images, i.e. chest/abdomen CT and brain MRI-based restaging every 6-12 weeks, without formal RECIST reevaluation, as several studies have demonstrated very good agreement between real-world and RECIST-based assessments (22, 23). OS was calculated from start of treatment for stage IV disease. Follow-up time was calculated by the reverse Kaplan-Meier method (24). Survival data were analyzed according to Kaplan-Meier and compared between patient groups with the logrank test. Numerical data were analyzed with the Student’s t-test, categorical data with the chi-square test, correlations with the Spearman’s coefficient, and effects of variables on survival were quantified by Cox regression. The multivariable Cox regression models were built with backward selection based on likelihood ratios using parameters significant in univariable testing. Confidence intervals for proportions were computed according to Clopper-Pearson (25). The rate of subsequent treatment across treatment lines for platinum- and non-platinum-based regimens was compared with a mixed linear model with the type of regimen (platinum-based or not) as fixed, and the treatment line as random variable. Statistical calculations were performed with SPSS v24 (IBM, Armonk, NY, USA), and plots generated with GraphPad Prism v9 (La Jolla, CA, USA).

### Ethics

This study was approved by the ethics committee of Heidelberg University (S-145/2017). Since this was a non-interventional, retrospective study, informed consent was obtained whenever possible, but its need for every participant was waived by the ethics committee.

## Results

### Baseline Patient Characteristics

**Figure 1:**
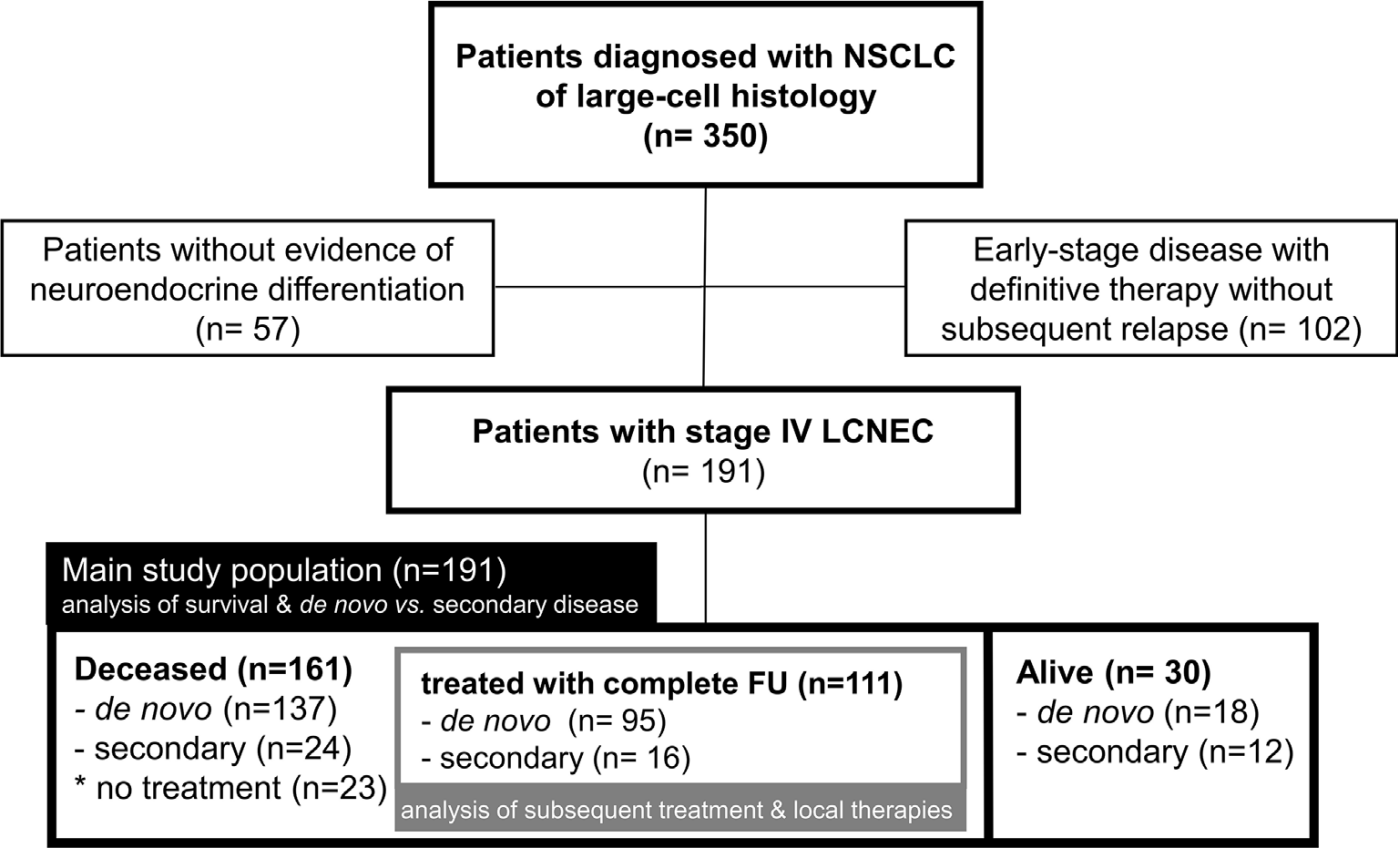
Flowchart of study patients and the populations used in each analysis. FU, follow-up.

Overall, 191 patients with stage IV LCNEC were identified (**Figure 1**). Among these, 155 patients (81%) were diagnosed with *de novo* (primary) metastatic disease, while 36 (19%) developed secondary stage IV LCNEC upon relapse or progression of previous early-stage tumors after definitive local treatment. At the time of data cut-off, 161 patients had died, among which 111 had received palliative systemic treatment with complete follow-up until death available (**Figure 1**). The characteristics of evaluable patients are summarized in **Table 1** and were very similar between the entire study population and the subset of deceased patients. Patient were mostly male (68%) current or former smokers (97%, 169/175), with a median age of 65 years (range 38 – 90) and a predominant baseline ECOG performance status (PS) of 0-1 (95%, **Table 1**). All patients showed immunohistochemical positivity for at least one of the following neuroendocrine markers: CD56 (156/170 tested patients), chromogranin A (74/119 tested patients), and synaptophysin (152/174 tested patients). PD-L1 TPS was available for 41 cases (1 case >50%, 21 cases 1-49%, 19 cases <1%; median 1%).

**Table 1:** Patient characteristics.

Clinical characteristics at diagnosis of stage IV and treatment		All study patients (n=191)		Deceased, treated with complete FU (n=111)	Secondary stage IV (n=36)
**Clinical characteristics at diagnosis of stage IV**					
Age, median (IQR)		65 (14)		64 (12)	63 (15)
Gender, % female (n)		32 (62)		37 (41)	28 (10)
Never/light smokers, % (n) ^1,2^		5 (9)		3 (3)	0 (0)
ECOG PS, % (n) ^1^	0	44.4 (76)		41.2 (42)	60 (21)
	1	50.3 (86)		54.9 (56)	36.4 (12)
	2	2.3 (4)		2 (2)	0 (0)
	3+	2.9 (5)		2 (2)	3 (1)
SCS, median (SD) ^1^		8 (2.98)		8 (2.79)	8 (2.94)
Metastatic sites at diagnosis, median (range)		1 (1-6)		2 (1-6)	1 (1-4)
Serum LDH (U/l), median (range) ^1^		271 (15-2594)		252 (15-2472)	247 (144-760)
Serum NSE (μg/l), median (range) ^1^		32 (10-492)		32 (10-361)	27 (11-85)
Blood NLR, median (range) ^1^		4.6 (0.2-58.6)		4.7 (0.2-58.6)	6.9 (1.4-20.7)
Serum CYFRA 21-1 (μg/l), median (range) ^1^		3.2 (0.8-969)		3.1 (0.8-969)	3.1 (0.8-13.9)
Serum CEA (μg/l), median (range) ^1^		3.8 (0.2-2678)		4.0 (0.3-1000)	4.3 (0.5-1000)
Any systemic treatment for stage IV disease					
- platinum-doublet in the first line, % (n) ^3^		74 (141/191)		86 (95/111)	56 (20/36)
- other chemotherapy in the first line ^4^		89 (17/191)		14 (15/111)	3 (1/36)
- platinum-doublet in any line, % (n)		75 (143/191)		87 (97/111)	56 (20/36)
- immunotherapy in any line, % (n) ^5^		7 (13/191)		7 (8/111)	11 (4/36)
- targeted therapies, % (n) ^6^		1 (2/191)		1 (1/111)	0 (0/36)
Local therapies in any line, % (n)		53 (101/191)		64 (71/111)	67 (24/36)
- for oligometastatic disease, % (n)		10 (9/92)		12 (8/65)	0 (0/23)
- palliative in the first line, % (n) ^7^		90 (83/92)		88 (57/65)	100 (23/23)
- palliative in any line, % (n) ^7^		91 (92/101)		89 (63/71)	100 (24/24)
Total number of treatment lines, median (range)		1 (0-7)		1 (1-7)	1 (0-4)
Follow-up time in months, median (95% CI)		42.7 (29.9-55.6)	n/a		42.7 (18.2-67.3)

IQR, interquartile range; PS, performance status; FU, follow-up; SCS, simplified comorbidity score; SD, standard deviation; NLR, neutrophil-to-lymphocyte ratio; 95% CI, 95% confidence interval.

^1^ smoking status for 175/191, 100/111, and 35/36 cases; ECOG PS status available for 171/191, 102/111, 33/36 SCS for 146/171, 99/111, 35/36; serum LDH for 152/191, 98/111, 22/36; blood NLR for 163/191, 101/111, 21/36; NSE for 146/191, 99/111, 21/36; CYFRA 21-1 for 136/191, 89/111, 17/36; CEA for 142/191, 92/111, 19/36.

^2^ never/light-smoking status refers to < 10 pack-years cumulatively.

^3^ etoposide (n=77), paclitaxel (n=54), gemcitabine (n=4), pemetrexed (n=1), vinorelbine (n=5).

^4^ vincristine/etoposide (n= 15), topotecan (n=1), docetaxel (n=1).

^5^ carboplatin-etoposide-atezolizumab (n=2), pembrolizumab (n=2), nivolumab (n=9).

^6^ one patient with an ALK fusion (26), and one patient with a RET fusion.

^7^ details about local therapies for patients with systemic treatment and complete follow-up are given in **Figure 7A**.

### Progression-Free and Overall Survival

**Figure 2:**
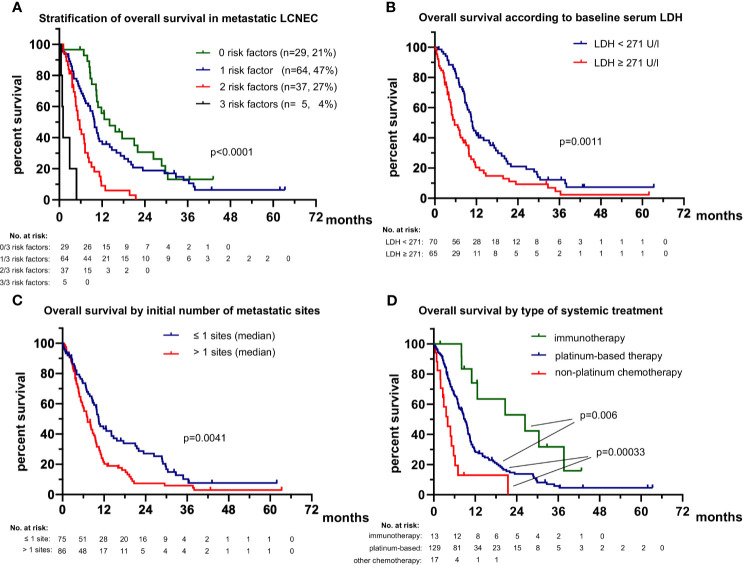
Overall survival of patients with metastatic LCNEC. **(A)** The median overall survival was 14.1 (95% confidence interval 7.9-20.3), 9.9 (8.6-11.2), 5.7 (4.3-7.1) and 1.1 (0.6-1.6) months (p < 0.001) for patients with 0, 1, 2 or 3 risk factors (lack of highly active systemic treatment with platinum and/or immunotherapy and/or TKI, baseline LDH ≥271 U/l, >1 initial metastatic sites; see **Table 2**). All patients with available values for all three parameters were included in this analysis (n = 135). **(B)** The median overall survival (OS) was 5.7 months (3.2-8.2) for patients with diagnosis of stage IV and serum LDH equal to or above the median for the entire study population (271 U/l, **Table 1**) *vs.* 10.9 months (9.9-11.9) for patients with diagnosis of stage IV and serum LDH below the median. **(C)** The median overall survival (OS) was 10.4 (8.5-12.3) months for patients with diagnosis of stage IV and ≤ 1 metastatic sites *vs.* 7.2 (5.3-10.0) months for patients with > 1 metastatic sites. **(D)** The median overall survival (OS) was 26.4 (6.4-46.5) months for patients who received immunotherapy in any treatment line *vs.* 9.0 (7.5-10.2) months for non-immunotherapy-treated patients, who received first-line platinum-based chemotherapy (logrank p=0.006 against immunotherapy) *vs.* 4.0 (1.6-6.4) months for patients who received other first-line chemotherapies (logrank p = 0.00033 against platinum-based chemotherapy). The rare (n = 2) TKI-treated patients were excluded from this analysis.

The rare patients (2/191 or 1%) with actionable genetic alterations (one with *ALK*, and one with *RET* fusion, **Table 1**) had a favorable course under TKI (death after 38 months of treatment with several ALK inhibitors as well as chemotherapy, partly published (26), and still alive with ongoing tumor response to second-line pralsetinib at 16.2 months, respectively). In order to identify independent determinants of clinical outcome in the vast majority of patients without routinely treatable mutations, we systematically analyzed the relationship of patient characteristics with PFS and OS (**Table 2**). In multivariable analysis, platinum-based chemotherapy, a lower number of metastatic sites at diagnosis of stage IV (≤1 sites), and additional local treatment (surgery or radiotherapy) were associated with significantly longer first-line PFS (hazard ratio [HR] 0.29 with p<0.001, HR 0.65 with p=0.035, and HR 0.64 with p=0.026, respectively). Besides, platinum-based first-line treatment (HR 0.20, p<0.001), administration of immunotherapy in any treatment line (HR 0.39, p=0.034), lower serum LDH (*i.e.* below the median of 271 U/l shown in **Table 1**, HR 0.54 with p=0.008), and a lower number of metastatic sites at diagnosis of stage IV (≤1 site, HR 0.52 with p=0.006) were independently associated with a longer OS from start of palliative systemic treatment (**Table 2**). Presence of 0 (n=29), 1 (n=64), 2 (n=37) or 3 (n=35) of the risk factors identified in multivariable analysis (type of systemic treatment, baseline LDH levels, number of initial metastatic sites, **Table 2**) could segregate patient survival with median OS values of 14.1, 9.9, 5.7 and 1.1 months, respectively (p<0.0001, **Figure 2**). For the smaller subset of patients who received immunotherapy in any line (n=13), median OS was even longer with 26.4 months *vs.* 9.0 months (p=0.006) for non-immunotherapy-treated patients who received first-line platinum-based chemotherapy *vs.* 4.0 for patients with non-platinum based first-line chemotherapies (p<0.001, **Figure 2D**). Immunotherapy was administered in combination with platinum-etoposide in the first line (n=2, atezolizumab), or as monotherapy in the first (n=1, pembrolizumab) or subsequent lines (n=8 nivolumab, n=1 pembrolizumab, n=1 atezolizumab). Taken together, the type of systemic treatment appeared to be the single most important factor for longer patient survival. Of note, neither the type of platinum drug, *i.e.* carboplatin *vs.* cisplatin, nor the partner cytostatic in the platinum-doublet (etoposide *vs.* paclitaxel, pemetrexed, gemcitabine or vinorelbine) appeared to have a significant effect on OS or PFS (p≥0.30, **Table 2**). First-line PFS was not reached for patients with first-line immunotherapy (n=3) *vs.* 4.6 months for other patients who received first-line platinum-based chemotherapy (p=0.051) *vs.* 1.5 months for patients who received other chemotherapies (p<0.001). The disease control rate (DCR) was significantly higher for patients treated with platinum-based compared to other chemotherapies (79% *vs.* 53%, p=0.028), while the objective response rate (ORR) was also numerically higher, but did not reach statistical significance (36% *vs.* 13%, p=0.078).

**Table 2:** Progression-free and overall survival of patients with metastatic LCNEC without treatable mutations.

	Univariable 1L PFS HR (95% CI)	Multivariable 1L PFS HR (95% CI)	Univariable OS HR (95% CI)	Multivariable OS HR (95% CI)
Age (</≥ 65)	0.85 (0.61-1.20) p=0.37		**0.64 (0.45-0.90) p=0.01**	—
Sex (male)	0.90 (0.63-1.28) p=0.54		0.63 0.64-1.31 p=0.63	
Smoker status (≥ 10 pack-years)	0.90 (0.41-1.95) p=0.79		0.77 (0.31-1.89) p=0.57	
ECOG performance status	1.30 (0.94-1.78) p=0.11		**1.75 (1.30-2.35) p<0.001**	—
*De novo vs.* secondary metastatic disease	0.78 (0.56-1.54) p=0.78		**0.56 (0.33-0.95) p=0.033**	—
Number of metastatic sites (≤/>1)	**0.68 (0.48-0.96) p=0.030**	**0.65 (0.44-0.97) p=0.035**	**0.61 (0.43-0.86) p=0.005**	**0.52 (0.33-0.83) p=0.006**
Presence of brain metastases	0.87 (0.61-1.26) p=0.47		1.14 (0.80-1.63) p=0.46	
SCS (≤/>8)	0.70 (0.47-1.04) p=0.08		1.28 (0.86-1.91) p=0.22	
Platinum-based first line	**0.31 (0.18-0.54) p<0.001**	**0.29 (0.15-0.54) p<0.001**	**0.34 (0.19-0.60) p<0.001**	**0.20 (0.10-0.42) p<0.001**
Platinum compound (cisplatin *vs.* carboplatin)	0.72 (0.38-1.35) p=0.30		0.72 (0.39-1.31) p=0.28	
Platinum partner (etoposide *vs.* others ^1^)	0.82 (0.56-1.20) p=0.30		1.04 (0.73-1.50) p=0.82	
Immunotherapy (*vs.* other systemic therapy) ^1^	n/a (3 cases)		**0.38 (0.18-0.77) p=0.007**	**0.39 (0.16-0.93) p=0.034**
Additional local therapy (radiotherapy/surgery ^2^)	**0.69 (0.49-0.98) p=0.035**	**0.64 (0.44-0.95) p=0.026**	**0.70 (0.50-0.98) p=0.038**	0.67 (0.43-1.04) p=0.08
Blood NLR (</≥ 5)	0.99 (0.69-1.45) p=0.99		**0.70 (0.49-0.99) p=0.049**	0.66 (0.43-1.01) p=0.052
Serum LDH (</≥ 271 U/l)	0.69 (0.48-1.01) p=0.057		**0.55 (0.38-0.79) p=0.001**	**0.54 (0.34-0.85) p=0.008**
Serum NSE (</≥ 32 μg/l)	**0.63 (0.43-0.92) p=0.017**	—	**1.59 (1.10-0.31) p=0.013**	—
Serum CYFRA (</≥ 4 μg/l)	0.77 (0.51-1.14) p=0.19		0.84 (0.57-1.24) p=0.39	
Serum CEA (</≥ 4 μg/l)	0.85 (0.58-1.25) p=0.42		0.95 (0.65-1.38) p=0.77	

1L, first line; SCS, simplified comorbidity score; n/a, not applicable.

^1^ for details please see footnote 3 and 5 of **Table 1**; ^2^ details about local therapies for patients with systemic treatment and complete follow-up are given in **Figure 7A**; for the PFS analysis, only local therapies during the 1L were considered, while for the OS analysis local therapies at any line were considered.

The relationship of various parameters shown in **Table 1** with progression-free survival (PFS) under first-line treatment, and overall survival (OS) was analyzed using a Cox regression. Continuous variables were dichotomized at the median values for the entire population (**Table 1**). Hazard ratios (HR) are given with 95% confidence intervals (CI) and p-values. The multivariable models were built with backward selection based on likelihood ratios using all parameters significant in univariable testing. Statistically significant results are highlighted in bold.

**Figure 3:**
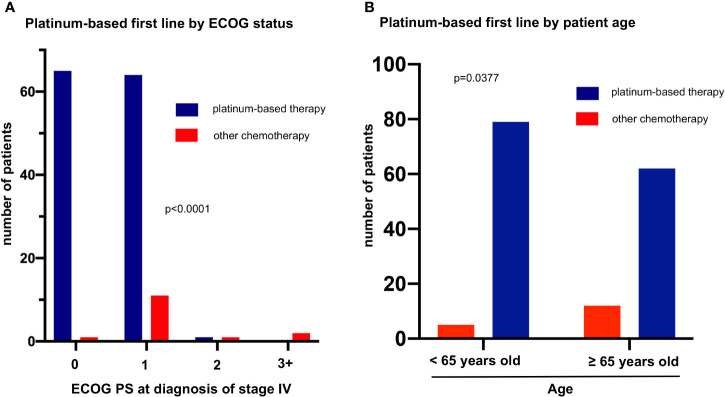
Factors associated with platinum-based first line in metastatic LCNEC. **(A)** The rate of platinum-based first-line treatment was 99% (65/66), 85% (64/75), 50% (1/2), and 0% (0/2) among patients with ECOG performance status (PS) 0, 1, 2, and ≥3 (chi-square p = 0.00004). **(B)** The rate of platinum-based first line treatment was 94% (79/84) among patients younger *vs.* 84% (141/158) among patients older than 65 years (median age, **Table 1**, chi-square p = 0.038).

Baseline patient characteristics directly associated with platinum administration in the first line were a better baseline ECOG PS (129/141 first-line patients with ECOG PS 0-1 received platinum *vs.* 1/4 with ECOG ≥2, p<0.001) and younger age below the median value of 65 years for the entire study population (79/84 platinum-treated patients with age <65 *vs.* 62/74 platinum-treated older patients, p=0.038, **Figure 3**). Both patient age and ECOG PS were significantly associated with OS only in univariable, but not in multivariable testing (**Table 2**), which suggests their relationship with patient survival is indirect, due to their influence on whether platinum-based treatment can be administered or not. A better baseline ECOG PS was significantly associated with fewer comorbidities, measured as a lower simplified comorbidity score (SCS, r=0.28, p=0.002), and – to a lesser extent – with younger age (r=0.18, p=0.022). Of note, baseline serum levels of the tumor markers NSE, CYFRA 21-1, CEA, the blood neutrophil-to-lymphocyte ratio (NLR), smoking status, and presence of brain metastases at diagnosis were not significantly associated with either PFS or OS of patients with metastatic LCNEC (**Table 2**).

### *De Novo vs.* Secondary Metastatic LCNEC

**Figure 4:**
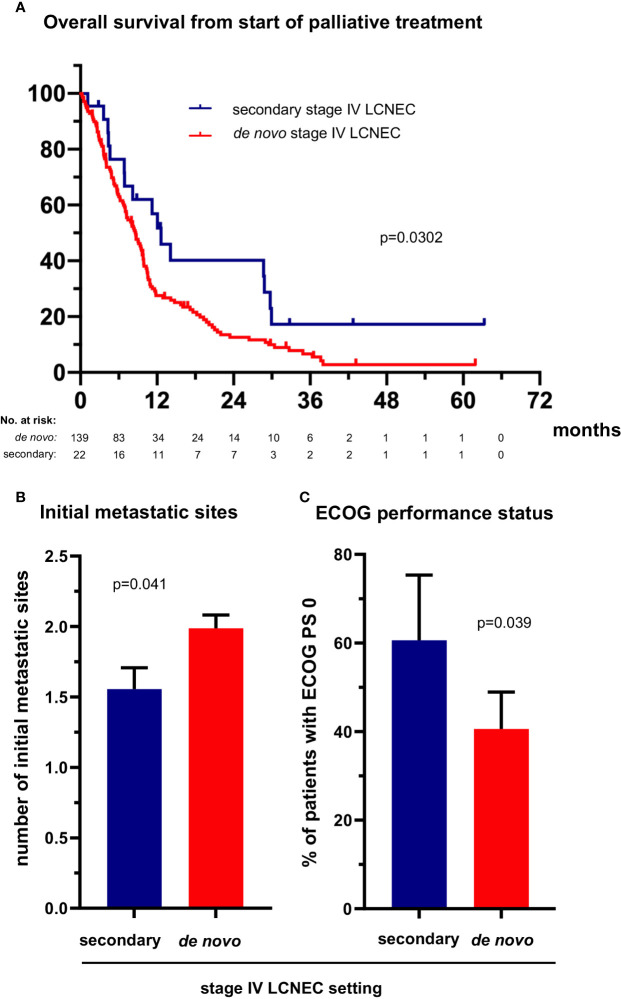
The clinical profile and survival of patients with secondary metastatic LCNEC. **(A)** The median overall survival from treatment start for stage IV disease was 12.6 (95% confidence interval [CI] 8.8-16.5) *vs.* 8.7 (6.9-10.4) months for patients with secondary *vs. de novo* metastatic LCNEC (logrank p = 0.030). **(B)** The median number of metastatic sites was 1.56 (standard error of the mean [SEM] 0.15) *vs.* 1.99 (SE 0.16, p = 0.041 with a t-test) for patients with secondary *vs. de novo* metastatic LCNEC. Error bars indicate SEM. **(C)** The percentage of cases with ECOG performance status (PS) 0 was 61% (20/33, CI 42-79) among patients with secondary *vs.* 41% (56/138, CI 32-49) among patients with *de novo* metastatic LCNEC (chi-square p = 0.038). Error bars indicate CI.

The 36 (19%) patients with secondary metastatic LCNEC (**Table 1**) had a longer OS from treatment start for metastatic disease than patients with *de novo* stage IV tumors (12.6 *vs.* 8.7 months in median, HR 0.56 with p=0.033, **Figure 4A** and **Table 2**). However, this association did not persist in multivariable testing (**Table 2**), and was therefore not direct, but possibly linked with the lower number of metastatic sites at diagnosis of secondary cases (average 1.6 vs. 2.0 for *de novo* cases, p=0.041, **Figure 4B**), which itself was an independent predictor of longer OS (**Table 2**). In addition, patients with secondary stage IV LCNEC had a better ECOG PS at diagnosis (ECOG 0 PS 61% *vs.* 39% for *de novo* cases, p=0.039, **Figure 4C**), which was associated with a higher likelihood of platinum-based therapy (**Figure 3**).

### Attrition Between Lines of Systemic Treatment for Metastatic LCNEC

**Figure 5:**
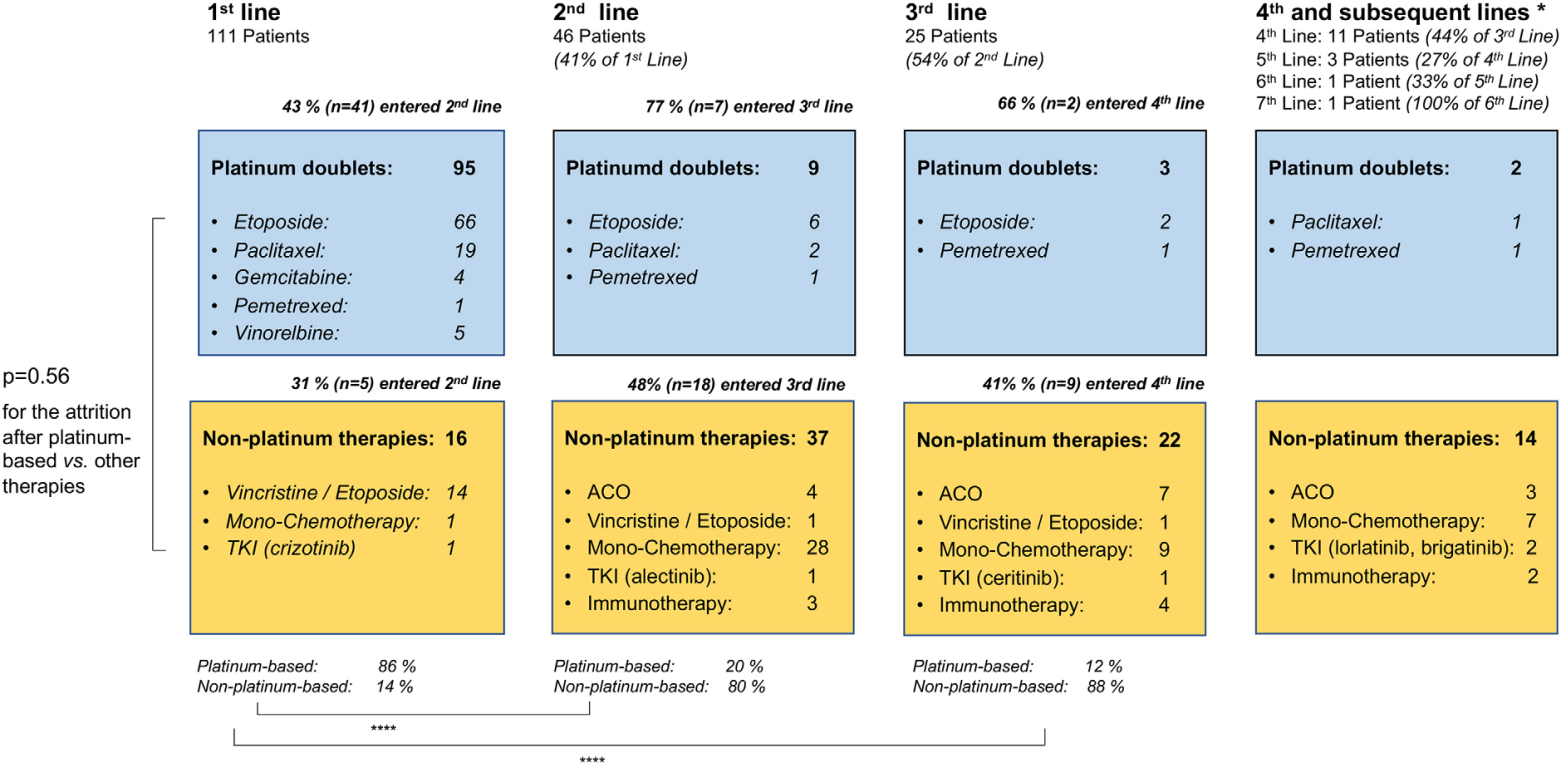
Flow of patients with metastatic LCNEC across treatment lines. This analysis was performed in the subset of patients who received palliative systemic treatment and have complete follow-up available until death (**Figure 1**). ACO, adriamycin, cyclophosphamide, vincristine; TKI, tyrosine kinase inhibitors; immunotherapy: immune checkpoint inhibitors. The ALK-positive LCNEC patient has been published previously (26). * 4th and subsequent lines cumulated; ****p < 0.0001 with a chi-square test for the percentage of platinum-based *vs.* non-platinum-based therapies in the first *vs.* second and third line; p = 0.56 with a mixed linear model for the attrition rate after platinum-based *vs.* other chemotherapies across the first three treatment lines.

**Figure 6:**
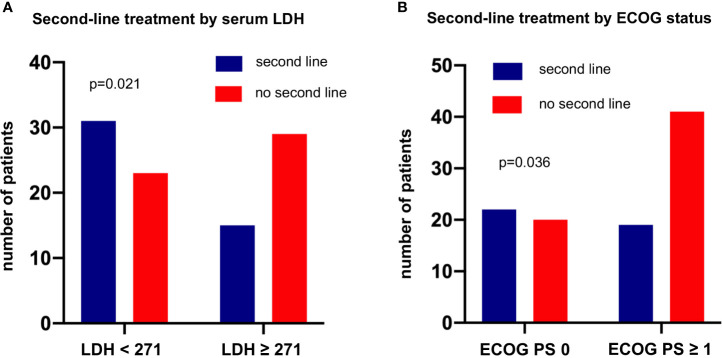
Baseline parameters at initial diagnosis associated with lack of subsequent treatment in metastatic LCNEC. This analysis was performed in the subset of patients who received palliative systemic treatment and have complete follow-up available until death (**Figure 1**). **(A)** The percentage of patients who missed second-line treatment was 66% (29/44) among patients with metastatic LCNEC with a higher serum LDH at diagnosis (≥ 271 U/l, i.e. above the median value, **Table 1**) *vs.* 43% (23/54) among patients with lower serum LDH levels (chi-square p = 0.0214). The LDH cut-off was based on the median value of the entire study population (**Table 1**). **(B)** The percentage of patients who missed second-line treatment was 68% (41/60) among patients with metastatic LCNEC and an ECOG performance status (PS) >0 at diagnosis *vs.* 48% (20/42) among patients with an ECOG PS 0 (chi-square p = 0.0357). The LDH and ECOG PS cut-offs were based on the median values of the entire study population (**Table 1**).

Among the entire study population, the percentage of patients who died without systemic treatment was 12% (23/191). Main reason was tumor-related clinical deterioration (n=13, 7%), while patient refusal of therapy (n=5) and severe concomitant illness (n=5, namely end-stage COPD in two cases, high-grade combined valvular heart disease, severe epilepsy, and psychosis requiring hospitalization) were less frequent. The median number of lines for palliative systemic treatment was 1 (range 1-7, **Table 1**). Among the 111 deceased patients with palliative systemic treatment and complete follow-up until death available (**Figure 1**), any second-line systemic treatment was administered to 41% (95% confidence interval [CI] 33%-51%, n=46, **Figure 5**). Between the second and third lines, a comparable attrition of 54% (25/46, CI 40%-68%) was observed (**Figure 5**). Platinum-based regimens dominated the first line (86% or 95/111) but were much less frequent subsequently (20% or 9/46 in the second, and 12% or 3/25 in the third line, p<0.001 compared to the first line, **Figure 5**). However, the attrition rate did not differ significantly for platinum *vs.* non-platinum-based regimens across the first 3 treatment lines (average rate of subsequent therapy 46.6% *vs.* 42.5%, respectively p=0.56 with a mixed linear model, as described in the Methods). Baseline parameters significantly associated with a lack of next-line treatment were higher serum LDH levels (66% *vs.* 43% for patients with serum LDH at diagnosis above or below the median, p=0.0214, **Figure 6A**), and a worse ECOG PS (68% *vs.* 48% for ECOG ≥1 *vs.* ECOG 0, chi-square p=0.036, **Figure 6B**). Along the same lines, the total number of chemotherapy lines administered to each patient correlated negatively with the baseline serum LDH levels (r=-0.22, p=0.03) and the ECOG PS at initial diagnosis (r=-0.20, p = 0.046).

### Utilization of Local Therapies and Relationship With Survival in Metastatic LCNEC

**Figure 7:**
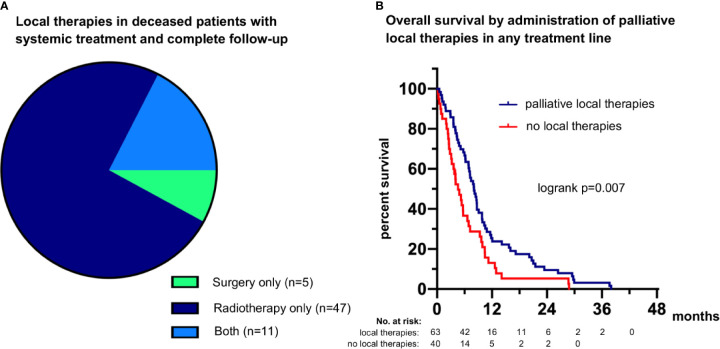
Palliative local therapies and their association with survival in metastatic LCNEC. **(A)** 75% of patients (47/63) received palliative radiotherapy only, 8% of patients (5/63) received palliative surgery, and 17% of patients (11/63) received both (**Table 1**). **(B)** The median overall survival (OS) was 8.1 (95% confidence interval 6.5-9.6) months for patients who received palliative local therapies during any treatment line *vs.* 6.9 (5.6-8.2) months for patients who did not (logrank p=0.007362).

Among the 111 deceased patients who received palliative systemic treatment and had complete follow-up until death (**Figure 1**), definitive local treatments in curative intention were administered to 8 for oligometastatic disease (7.2%), while 63 (57%) patients received palliative radiotherapy (n=47), palliative surgery (n=5), or both (n=11) in any therapy line (**Table 1** and **Figure 7A**). After exclusion of oligometastatic cases, administration of palliative local therapies at any time during systemic treatment correlated positively with OS (HR 0.58 with p=0.008, **Figure 7B**) and the total number of chemotherapy lines (r=0.27, p=0.004). However, the association between local therapies and OS did not persist with multivariable testing (**Table 2**) and was therefore probably not causal, but rather secondary due to the longer duration of treatment for LCNEC patients with better prognosis.

## Discussion

LCNEC is a rare pulmonary malignancy with poor prognosis and scarce evidence to guide management. These patients are underrepresented in clinical trials of unselected NSCLC, while published LCNEC series are either small, with generally fewer than 50-70 cases (27), or rely on the basic information available in national registers (28–32). Detailed capturing of heterogeneity across the entire clinical spectrum of LCNEC requires deep annotation of large patient numbers in order to provide insights useful for management of this challenging entity.

The single most important factor for longer patient survival in the vast majority of LCNEC patients without routinely treatable genetic alterations according to the results of the current study is highly active systemic treatment with administration of platinum-based chemotherapy and/or immunotherapy (**Table 2** and **Figure 2**). The type of systemic treatment had a stronger effect on OS and PFS than clinical disease parameters and laboratory results, like the metastatic pattern and serum levels of various tumor markers. In addition, the type of treatment emerged as more important for patient survival than patient-related characteristics, like age and ECOG PS, whose relationship with OS did not persist in multivariable testing (**Table 2**) and therefore likely results from their association with administration or not of platinum-based chemotherapy (**Figure 3**). The observed association between administration of local treatments and longer OS (**Figure 7**) is probably also secondary, due to the longer duration of treatment, which permits more ablative interventions across the entire disease trajectory, since this relationship did not persist in multivariable testing either (**Table 2**). On the other hand, the type of platinum drug (cisplatin or carboplatin) and the cytotoxic partner in the platinum doublet (etoposide *vs.* other NSCLC drugs) did not matter (**Table 2**). However, it should be noted here that our “NSCLC-drug” group included mainly patients with platinum/paclitaxel (n=54/64, footnote 3 of **Table 1**), so that putative equivalence with etoposide cannot be safely concluded for other chemotherapeutics, inasmuch a previous study has demonstrated inferiority of pemetrexed in this setting (33). The median PFS and OS of 4.6 and 9.0 months, respectively, observed for patients receiving platinum-based first-line therapy in this study (**Figure 2**) are comparable to the results of other prospective and retrospective LCNEC series analyzing platinum regimens, which have observed median PFS intervals of 4–6 and median OS intervals of 8–12 months (10, 34–37).

Of particular interest are the more favorable characteristics and course of LCNEC patients with secondary metastatic disease (**Figure 4**). This finding extends the observations of some older studies, which had observed a longer OS for chemotherapy-treated patients with secondary stage IV NSCLC of the usual histological subtypes (38–40). Special features of secondary metastatic lung cancer in the current and previous studies are a lower number of initial metastatic sites and a better baseline ECOG PS (38, 39) (**Figure 4**), parameters which are linked to a better OS of LCNEC patients (**Table 2**) and could explain the improved prognosis compared to *de novo* cases. These differences are likely caused by the fact that secondary metastatic disease is diagnosed earlier, during the routine follow-up after definitive treatment of the preceding early-stage tumors, which can detect subsequent relapse or progression already while this is still asymptomatic. Consistent with this notion, a more recent study analyzing tyrosine kinase inhibitor (TKI)-treated patients with *EGFR*-mutated NSCLC found very similar molecular profiles between *de novo* and secondary tumors, despite clinical differences similar to those observed between *de novo* and secondary stage IV LCNEC (41). Of note, the proportion of secondary metastatic LCNEC, namely 36/191 or 19% in this study, is not a biologic feature either, but depends on the efficacy of lung cancer detection strategies. For example, the upcoming adoption of CT-based lung cancer screening is expected to increase this ratio, since many tumors currently detected as *de novo* metastatic LCNEC will then be diagnosed already in earlier stages, and also proportionally contribute more to the pool of “secondary” cases after relapse (42).

The observed patient attrition rate between lines of systemic treatment observed in this study was approximately 50% (**Figure 5**), which is very similar to the rate reported for chemotherapy-treated NSCLC of the usual histological subtypes (43–45). Lack of subsequent treatment was significantly associated with more aggressive disease, evident as a higher baseline serum LDH (**Figure 6A**), and with a worse patient condition, as captured by a worse ECOG PS at diagnosis (**Figure 6B**), but did not differ according to the type of treatment (platinum-based or not, **Figure 5**). The lower rate of attrition reported for *EGFR*- and *ALK*-mutated NSCLC, i.e. approximately 30% in the real-world setting ( (46) and data of the authors about ALK^+^ NSCLC currently under review for publication), is probably attributable to the higher efficacy of targeted therapies and the lower biologic aggressiveness of these tumors (47, 48), as also suggested by the more frequent occurrence of oligoprogression in these tumors compared to non-oncogene-dependent NSCLC (49).

The clinical utility of TKI is very limited in LCNEC currently, because routinely actionable genetic alterations are exceedingly rare, <2% in the literature (7, 50) as well as our cohort (**Table 1**), and “blind” administration of targeted drugs without reliable predictive biomarkers is unlikely to have a significant impact (51). This underlines the importance of immunotherapy, for which a survival benefit of several months has recently been demonstrated in several retrospective series of LCNEC patients (15–18). In our study, the gain was also considerable, with a median OS from the start of palliative systemic treatment exceeding 2 years (**Figure 2A**). This result is well in line with the higher tumor mutational burden (TMB) of LCNEC compared to other lung cancers (52) and with the importance of PD-L1 expression on LCNEC cells and their microenvironment for patient outcome (53). Of special interest in this regard is also the exquisite immune reactivity of LCNEC, with readily detectable T-cell receptor (TCR) repertoire alterations in the peripheral blood, which could be exploited for guidance of immunotherapeutic interventions and improved disease monitoring (54).

Main limitation of our study is its retrospective design, which cannot guarantee absence of some confounding. In addition, delineating the diagnosis of LCNEC from the small biopsies routinely used in case of metastatic disease is challenging and cannot exclude presence of another NSCLC component outside the sampled area (3). Nevertheless, this might not have major clinical consequences, as mixed LCNEC tumors appear to have a similar aggressiveness and prognosis as their “pure” counterparts (4).

Also, while the number of immunotherapy-treated patients in our study was not large, our results add to the observations of previous studies which similarly demonstrated a significant benefit from immune checkpoint inhibitors in metastatic LCNEC (15–18). Beyond this, our work describes one of the largest published LCNEC cohorts, with homogenous decision-making in the same institution and deep clinical annotation, which make it a valuable source of insights into the contemporary clinical reality of this rare entity. The delineation of treatment patterns, elucidation of the complex interplay between clinicolaboratory parameters and the disease course, as well as the identification of factors with key importance for patient survival presented here, could support daily clinical practice and provide a benchmark for assessing the real-world impact of future therapeutic developments.

## Data Availability Statement

The raw data supporting the conclusions of this article will be made available by the authors, without undue reservation.

## Ethics Statement

The studies involving human participants were reviewed and approved by ethics committee of Heidelberg University S-145/2017. Written informed consent for participation was not required for this retrospective study in accordance with the national legislation and the institutional requirements.

## Author Contributions

DF: conceptualization, methodology, investigation, data curation, formal analysis, visualization, writing - original draft. FB: investigation, data curation, validation, writing - review & editing. DK: investigation, data curation, validation, writing – original draft. JK: investigation, data curation, validation, writing - review & editing. LK: investigation, data curation, validation, writing - review & editing. RS: investigation, data curation, validation, writing - review & editing. FE: investigation, data curation, validation, writing - review & editing. MK: investigation, data curation, writing - review & editing. MS: investigation, data curation, validation, supervision, writing - review & editing. TM: investigation, data curation, validation, supervision, writing - review & editing. AS: validation, supervision, project administration, writing - review & editing. HB: methodology, validation, writing - review & editing. PC: conceptualization, methodology, investigation, data curation, formal analysis, visualization, supervision, project administration, writing - original draft, writing - review & editing. All authors contributed to the article and approved the submitted version.

## Funding

This study was funded by the German Center for Lung Research (DZL). The funding source did not have any influence on the design, conduction, and report of the results for this study.

## Conflict of Interest

FB reports research funding from BMS and travel grants from BMS and MSD. DK reports advisory board and speaker’s honoraria from AstraZeneca, BMS, Pfizer. JK reports research funding from AstraZeneca and Celgene. RS reports research funding from BMS and speaker’s honoraria from Roche. TM reports research funding from Roche and patents with Roche. AS reports advisory board honoraria from BMS, AstraZeneca, ThermoFisher, Novartis, speaker’s honoraria from BMS, Illumina, AstraZeneca, Novartis, ThermoFisher, MSD, Roche, and research funding from Chugai. PC reports research funding from AstraZeneca, Novartis, Roche, Takeda, and advisory board/lecture fees from AstraZeneca, Boehringer Ingelheim, Chugai, Novartis, Pfizer, Roche, Takeda.

The remaining authors declare that the research was conducted in the absence of any commercial or financial relationships that could be construed as a potential conflict of interest.

## References

[1] Travis WD Linnoila RI Tsokos MG Hitchcock CL Cutler Jr GB Nieman L. Neuroendocrine Tumors of the Lung With Proposed Criteria for Large-Cell Neuroendocrine Carcinoma. An Ultrastructural, Immunohistochemical, and Flow Cytometric Study of 35 Cases. Am J Surg Pathol (1991) 15:529–53. 10.1097/00000478-199106000-00003 1709558

[2] Travis WD. Lung Tumours With Neuroendocrine Differentiation. Eur J Cancer (2009) 45:251–66. 10.1016/S0959-8049(09)70040-1 19775623

[3] Travis WD Brambilla E Nicholson AG Yatabe Y Austin JH Beasley MB. The 2015 World Health Organization Classification of Lung Tumors: Impact of Genetic, Clinical and Radiologic Advances Since the 2004 Classification. J Thor Oncol (2015) 10:1243–60. 10.1097/JTO.0000000000000630 26291008

[4] Battafarano RJ Fernandez FG Ritter J Meyers BF Guthrie TJ Cooper JD. Large Cell Neuroendocrine Carcinoma: An Aggressive Form of non-Small Cell Lung Cancer. J Thorac Cardiovasc Surg (2005) 130:166–72. 10.1016/j.jtcvs.2005.02.064 15999058

[5] Fasano M Della Corte CM Papaccio F Ciardiello F Morgillo F. Pulmonary Large-Cell Neuroendocrine Carcinoma: From Epidemiology to Therapy. J Thor Oncol (2015) 10:1133–41. 10.1097/JTO.0000000000000589 PMC4503246 26039012

[6] Deng C Wu S-G Tian Y. Lung Large Cell Neuroendocrine Carcinoma: An Analysis of Patients From the Surveillance, Epidemiology, and End-Results (SEER) Database. Med Sci Monit (2019) 25:3636–46. 10.12659/MSM.914541 PMC6537662 31095532

[7] Rossi G Cavazza A Marchioni A Longo L Migaldi M Sartori G. Role of Chemotherapy and the Receptor Tyrosine Kinases KIT, Pdgfralpha, PDGFRbeta, and Met in Large-Cell Neuroendocrine Carcinoma of the Lung. J Clin Oncol (2005) 23:8774–85. 10.1200/JCO.2005.02.8233 16314638

[8] George J Walter V Peifer M Alexandrov LB Seidel D Leenders F. Integrative Genomic Profiling of Large-Cell Neuroendocrine Carcinomas Reveals Distinct Subtypes of High-Grade Neuroendocrine Lung Tumors. Nat Commun (2018) 9:1048. 10.1038/s41467-018-03099-x 29535388 PMC5849599

[9] Glisson BS Moran CA. Large-Cell Neuroendocrine Carcinoma: Controversies in Diagnosis and Treatment. J Natl Compr Canc Netw (2011) 9:1122–9. 10.6004/jnccn.2011.0093 21975912

[10] Sun JM Ahn MJ Ahn JS Um SW Kim H Kim HK. Chemotherapy for Pulmonary Large Cell Neuroendocrine Carcinoma: Similar to That for Small Cell Lung Cancer or Non-Small Cell Lung Cancer? Lung Cancer (2012) 77:365–70. 10.1016/j.lungcan.2012.04.009 22579297

[11] Naidoo J Santos-Zabala ML Iyriboz T Woo KM Sima CS Fiore JJ. Large Cell Neuroendocrine Carcinoma of the Lung: Clinico-Pathologic Features, Treatment, and Outcomes. Clin Lung Cancer (2016) 17:e121–9. 10.1016/j.cllc.2016.01.003 PMC5474315 26898325

[12] Hiroshima K Mino-Kenudson M. Update on Large Cell Neuroendocrine Carcinoma. Transl Lung Cancer Res (2017) 6:530–9. 10.21037/tlcr.2017.06.12 PMC5653527 29114469

[13] Derks JL Leblay N Thunnissen E van Suylen RJ den Bakker M Groen HJ. Molecular Subtypes of Pulmonary Large-Cell Neuroendocrine Carcinoma Predict Chemotherapy Treatment Outcome. Clin Cancer Res (2018) 24:33–42. 10.1158/1078-0432.CCR-17-1921 29066508

[14] Rekhtman N Pietanza MC Hellmann MD Naidoo J Arora A Won H. Next-Generation Sequencing of Pulmonary Large Cell Neuroendocrine Carcinoma Reveals Small Cell Carcinoma-Like and Non-Small Cell Carcinoma-Like Subsets. Clin Cancer Res (2016) 22:3618–29. 10.1158/1078-0432.CCR-15-2946 PMC4995776 26960398

[15] Dudnik E Kareff S Moskovitz M Kim C Liu SV Lobachov A. Real-World Survival Outcomes With Immune Checkpoint Inhibitors in Large-Cell Neuroendocrine Tumors of Lung. J Immunother Cancer (2021) 9:e001999. 10.1136/jitc-2020-001999 33597218 PMC7893659

[16] Levra MG Mazieres J Valette CA Molinier O Planchard D Frappat V. P1.07-012 Efficacy of Immune Checkpoint Inhibitors in Large Cell Neuroendocrine Lung Cancer: Results From a French Retrospective Cohort. J Thor Oncol (2016) 12:S702–3. 10.1016/j.jtho.2016.11.923

[17] Sherman S Rotem O Shochat T Zer A Moore A Dudnik E. Efficacy of Immune Check-Point Inhibitors (ICPi) in Large Cell Neuroendocrine Tumors of Lung (LCNEC). Lung Cancer (2020) 143:40–6. 10.1016/j.lungcan.2020.03.008 32203769

[18] Komiya T Powell E. Role of Immunotherapy in Stage IV Large Cell Neuroendocrine Carcinoma of the Lung. J Clin Oncol (2020) 38:9060. 10.1200/JCO.2020.38.15_suppl.9060 PMC8190341 33639649

[19] Volckmar AL Leichsenring J Kirchner M Christopoulos P Neumann O Budczies J. Combined Targeted DNA and RNA Sequencing of Advanced NSCLC in Routine Molecular Diagnostics: Analysis of the First 3,000 Heidelberg Cases. Int J Cancer (2019) 145:649–61. 10.1002/ijc.32133 30653256

[20] Planchard D Popat S Kerr K Novello S Smit EF Faivre-Finn C. Metastatic Non-Small Cell Lung Cancer: ESMO Clinical Practice Guidelines for Diagnosis, Treatment and Follow-Up. Ann Oncol (2018) 29:iv192–237. 10.1093/annonc/mdy275 30285222

[21] Colinet B Jacot W Bertrand D Lacombe S Bozonnat MC Daurès JP. A New Simplified Comorbidity Score as a Prognostic Factor in non-Small-Cell Lung Cancer Patients: Description and Comparison With the Charlson’s Index. Br J Cancer (2005) 93:1098–105. 10.1038/sj.bjc.6602836 PMC2361505 16234816

[22] Ma X Nussbaum NC Magee K Bourla AB Tucker M Bellomo L. Comparison of Real-World Response Rate (rwRR) to RECIST-Based Response Rate in Patients With Advanced non-Small Cell Lung Cancer (aNSCLC). Ann Oncol (2019) 30:1581P. 10.1093/annonc/mdz260.103

[23] Bartlett CH Mardekian J Cotter M Huang X Zhang Z Parrinello CM. Concordance of Real World Progression Free Survival (PFS) on Endocrine Therapy as First Line Treatment for Metastatic Breast Cancer Using Electronic Health Record With Proper Quality Control Versus Conventional PFS From a Phase 3 Trial. Cancer Res (2018) 78:P3–17-03. 10.1158/1538-7445.Sabcs17-p3-17-03

[24] Schemper M Smith TL. A Note on Quantifying Follow-Up in Studies of Failure Time. Control Clin Trials (1996) 17:343–6. 10.1016/0197-2456(96)00075-x 8889347

[25] Clopper CJ Pearson ES. The Use of Confidence or Fiducial Limits Illustrated in the Case of the Binomial. Biometrika (1934) 26:404–13. 10.2307/2331986

[26] Wiedemann C Kunz J Kirchner M Volckmar A-L Winter H Bochtler T. A Rare Case of Large-Cell Neuroendocrine Lung Carcinoma Sensitive to ALK Inhibitors. Cancer Res Treat (2019) 42:210. 10.1159/000502425

[27] Lo Russo G Pusceddu S Proto C Macerelli M Signorelli D Vitali M. Treatment of Lung Large Cell Neuroendocrine Carcinoma. Tumour Biol (2016) 37:7047–57. 10.1007/s13277-016-5003-4 26943800

[28] Derks JL Hendriks LE Buikhuisen WA Groen HJ Thunnissen E van Suylen RJ. Clinical Features of Large Cell Neuroendocrine Carcinoma: A Population-Based Overview. Eur Respir J (2016) 47:615–24. 10.1183/13993003.00618-2015 26541538

[29] Rossi G Mengoli MC Cavazza A. Pulmonary Large Cell Neuroendocrine Carcinoma: A True High-Grade Neuroendocrine Tumor Needing Prospective Therapeutic Data. J Thorac Oncol (2011) 6:1775. 10.1097/JTO.0b013e31822a3658 21918393

[30] Ioannidis G. Pulmonary Large-Cell Neuroendocrine Carcinoma: Therapeutic Challenges and Opportunities. Forum Clin Oncol (2020) 11:7–21. 10.2478/fco-2019-0010

[31] Kinslow CJ May MS Saqi A Shu CA Chaudhary KR Wang TJ. Large-Cell Neuroendocrine Carcinoma of the Lung: A Population-Based Study. Clin Lung Cancer (2020) 21:e99–e113. 10.1016/j.cllc.2019.07.011 31601526

[32] Cao L Li Z-W Wang M Zhang T-T Bao B Liu Y-P. Clinicopathological Characteristics, Treatment and Survival of Pulmonary Large Cell Neuroendocrine Carcinoma: A SEER Population-Based Study. PeerJ (2019) 7:e6539. 10.7717/peerj.6539 30944773 PMC6441320

[33] Derks JL van Suylen RJ Thunnissen E den Bakker MA Groen HJ Smit EF. Chemotherapy for Pulmonary Large Cell Neuroendocrine Carcinomas: Does the Regimen Matter? Eur Respir J (2017) 49(6):1601838. 10.1183/13993003.01838-2016 28572122 PMC5898951

[34] Le Treut J Sault MC Lena H Souquet PJ Vergnenegre A Le Caer H. Multicentre Phase II Study of Cisplatin-Etoposide Chemotherapy for Advanced Large-Cell Neuroendocrine Lung Carcinoma: The GFPC 0302 Study. Ann Oncol (2013) 24:1548–52. 10.1093/annonc/mdt009 23406729

[35] Niho S Kenmotsu H Sekine I Ishii G Ishikawa Y Noguchi M. Combination Chemotherapy With Irinotecan and Cisplatin for Large-Cell Neuroendocrine Carcinoma of the Lung: A Multicenter Phase II Study. J Thorac Oncol (2013) 8:980–4. 10.1097/JTO.0b013e31828f6989 23774385

[36] Fujiwara Y Sekine I Tsuta K Ohe Y Kunitoh H Yamamoto N. Effect of Platinum Combined With Irinotecan or Paclitaxel Against Large Cell Neuroendocrine Carcinoma of the Lung. Jpn J Clin Oncol (2007) 37:482–6. 10.1093/jjco/hym053 17652109

[37] Tokito T Kenmotsu H Watanabe R Ito I Shukuya T Ono A. Comparison of Chemotherapeutic Efficacy Between LCNEC Diagnosed Using Large Specimens and Possible LCNEC Diagnosed Using Small Biopsy Specimens. Int J Clin Oncol (2014) 19:63–7. 10.1007/s10147-012-0509-2 23250620

[38] Moore S Leung B Wu J Ho C. Survival Implications of De Novo Versus Recurrent Metastatic non-Small Cell Lung Cancer. Am J Clin Oncol (2019) 42:292–7. 10.1097/COC.0000000000000513 30608237

[39] Gibson AJ Li H D’Silva A Tudor RA Elegbede AA Otsuka S. Comparison of Clinical Characteristics and Outcomes in Relapsed Versus De Novo Metastatic non-Small Cell Lung Cancer. Am J Clin Onc (2019) 42:75–81. 10.1097/COC.0000000000000483 30211724

[40] Yu HA Sima CS Hellmann MD Naidoo J Busby N Rodriguez K. Differences in the Survival of Patients With Recurrent Versus De Novo Metastatic KRAS-Mutant and EGFR-Mutant Lung Adenocarcinomas. Cancer (2015) 121:2078–82. 10.1002/cncr.29313 PMC4783794 25781862

[41] Bozorgmehr F Kazdal D Chung I Kirchner M Magios N Kriegsmann K. De Novo Versus Secondary Metastatic EGFR-Mutated non-Small-Cell Lung Cancer. Front Oncol (2021) 11:640048. 10.3389/fonc.2021.640048 33898315 PMC8063726

[42] de Koning HJ van der Aalst CM de Jong PA Scholten ET Nackaerts K Heuvelmans MA. Reduced Lung-Cancer Mortality With Volume CT Screening in a Randomized Trial. N Engl J Med (2020) 382:503–13. 10.1056/NEJMoa1911793 31995683

[43] Sacher AG Le LW Lau A Earle CC Leighl NB. Real-World Chemotherapy Treatment Patterns in Metastatic non-Small Cell Lung Cancer: Are Patients Undertreated? Cancer (2015) 121:2562–9. 10.1002/cncr.29386 25891153

[44] Zietemann V Duell T. Every-Day Clinical Practice in Patients With Advanced Non-Small-Cell Lung Cancer. Lung Cancer (2010) 68:273–7. 10.1016/j.lungcan.2009.06.023 19632737

[45] Zietemann V Duell T. Prevalence and Effectiveness of First-, Second-, and Third-Line Systemic Therapy in a Cohort of Unselected Patients With Advanced non-Small Cell Lung Cancer. Lung Cancer (2011) 73:70–7. 10.1016/j.lungcan.2010.10.017 21095039

[46] Magios N Bozorgmehr F Volckmar AL Kazdal D Kirchner M Herth F. Real-World Implementation of Sequential Targeted Therapies for EGFR-Mutated Lung Cancer. Ther Adv Med Oncol (2021) 13:1–13. 10.1177/1758835921996509 PMC8366107 34408792

[47] Christopoulos P Kirchner M Roeper J Saalfeld F Janning M Bozorgmehr F. Risk Stratification of EGFR+ Lung Cancer Diagnosed With Panel-Based Next-Generation Sequencing. Lung Cancer (2020) 148:105–12. 10.1016/j.lungcan.2020.08.007 32871455

[48] Christopoulos P Budczies J Kirchner M Dietz S Sultmann H Thomas M. Defining Molecular Risk in ALK(+) NSCLC. Oncotarget (2019) 10:3093–103. 10.18632/oncotarget.26886 PMC6517105 31139322

[49] Rheinheimer S Heussel C-P Mayer P Gaissmaier L Bozorgmehr F Winter H. Oligoprogressive Non-Small-Cell Lung Cancer Under Treatment With PD-(L)1 Inhibitors. Cancers (Basel) (2020) 12(4):1046. 10.3390/cancers12041046 PMC7226015 32340408

[50] Miyoshi T Umemura S Matsumura Y Mimaki S Tada S Makinoshima H. Genomic Profiling of Large-Cell Neuroendocrine Carcinoma of the Lung. Clin Cancer Res (2017) 23:757–65. 10.1158/1078-0432.CCR-16-0355 27507618

[51] Christopoulos P Engel-Riedel W Grohe C Kropf-Sanchen C von Pawel J Gutz S. Everolimus With Paclitaxel and Carboplatin as First-Line Treatment for Metastatic Large-Cell Neuroendocrine Lung Carcinoma: A Multicenter Phase II Trial. Ann Oncol (2017) 28:1898–902. 10.1093/annonc/mdx268 28535181

[52] Sabari JK Julian RA Ni A Halpenny D Hellmann MD Drilon AE. Outcomes of Advanced Pulmonary Large Cell Neuroendocrine Carcinoma Stratified by RB1 Loss, SLFN11 Expression, and Tumor Mutational Burden. J Clin Oncol (2018) 36:e20568–8. 10.1200/JCO.2018.36.15_suppl.e20568

[53] Eichhorn F Harms A Warth A Muley T Winter H Eichhorn ME. PD-L1 Expression in Large Cell Neuroendocrine Carcinoma of the Lung. Lung Cancer (2018) 118:76–82. 10.1016/j.lungcan.2018.02.003 29572007

[54] Christopoulos P Schneider MA Bozorgmehr F Kuon J Engel-Riedel W Kollmeier J. Large Cell Neuroendocrine Lung Carcinoma Induces Peripheral T-Cell Repertoire Alterations With Predictive and Prognostic Significance. Lung Cancer (2018) 119:48–55. 10.1016/j.lungcan.2018.03.002 29656752

